# TASL mediates keratinocyte differentiation by regulating intracellular calcium levels and lysosomal function

**DOI:** 10.1038/s41598-024-61674-3

**Published:** 2024-05-14

**Authors:** Ji Yeong Park, Hyeng-Soo Kim, Hyejin Hyung, Soyeon Jang, Jiwon Ko, Jin Hong Lee, Si-Yong Kim, Song Park, Junkoo Yi, Sijun Park, Su-Geun Lim, Seonggon Kim, Sanggyu Lee, Myoung Ok Kim, Soyoung Jang, Zae Young Ryoo

**Affiliations:** 1 School of Life Sciences, BK21 FOUR KNU Creative BioResearch Group, Kyungpook National University, Daegu, 41566 Republic of Korea; 2 Institute of Life Science and Biotechnology, Kyungpook National University, Daegu, 41566 Republic of Korea; 3 Division of Animal Science, Gyeongsang National University, Jinju, 52828 Republic of Korea; 4 Institute of Agriculture and Life Science (IALS), Gyeongsang National University, Jinju, 52828 Republic of Korea; 5 School of Animal Life Convergence Science, Hankyong National University, Anseong, 17579 Republic of Korea; 6 Preclinical Research Center, Daegu-Gyeongbuk Medical Innovation Foundation, Daegu, Republic of Korea; 7 Department of Animal Science and Biotechnology, Research Institute for Innovative Animal Science, Kyungpook National University, Sangju-si, Gyeongsang buk-do 37224 Republic of Korea

**Keywords:** Cell biology, Diseases

## Abstract

Maintaining epidermal homeostasis relies on a tightly organized process of proliferation and differentiation of keratinocytes. While past studies have primarily focused on calcium regulation in keratinocyte differentiation, recent research has shed light on the crucial role of lysosome dysfunction in this process. TLR adaptor interacting with SLC15A4 on the lysosome (TASL) plays a role in regulating pH within the endo-lysosome. However, the specific role of TASL in keratinocyte differentiation and its potential impact on proliferation remains elusive. In our study, we discovered that TASL deficiency hinders the proliferation and migration of keratinocytes by inducing G1/S cell cycle arrest. Also, TASL deficiency disrupts proper differentiation process in TASL knockout human keratinocyte cell line (HaCaT) by affecting lysosomal function. Additionally, our research into calcium-induced differentiation showed that TASL deficiency affects calcium modulation, which is essential for keratinocyte regulation. These findings unveil a novel role of TASL in the proliferation and differentiation of keratinocytes, providing new insights into the intricate regulatory mechanisms of keratinocyte biology.

## Introduction

The skin consists of two main layers, the epidermis and dermis^1^. The epidermis serves as a protective barrier against external factors, maintaining homeostasis through constant proliferation and differentiation. It has four layers: stratum basale, stratum spinosum, stratum granulosum, and stratum corneum. The epidermis is mainly composed of keratinocytes, which proliferate in the basal layer and then differentiate as they move towards the stratum corneum, the outermost layer. Keratinocytes constantly replenish themselves through proliferation and differentiation to maintain homeostasis^2^. When the formation of the epidermal barrier or keratinocyte differentiation is compromised, tissue integrity is disrupted, making it more susceptible to mechanical stress and penetration by pathogens. This breakdown of epidermal homeostasis can lead to conditions such as Darier disease or epidermolytic ichthyosis. In addition, it can be a contributing factor in conditions such as eczema or contact dermatitis^3,4^.

Calcium ion serves as an internal second messenger that regulates various cellular processes^5^. Ca^2+^ signaling is essential for regulating the proliferation, differentiation, cell-to-cell adhesion, migration, and apoptosis of keratinocytes^6,7^. In response to elevated calcium levels, keratinocytes differentiate and sequentially express Cytokeratin 10 (CK10), Involucrin, and Filaggrin. Calcium-sensing receptor (CaSR) detects extracellular calcium and activates phospholipase C (PLC), resulting in the hydrolysis of phosphatidylinositol-4,5-bisphosphate (PIP2) into inositol-1,4,5-trisphosphate (IP3) and diacylglycerol (DAG). These signaling cascades promote cell-cell adhesion, cell survival, and differentiation^8^. In addition, IP3 releases calcium from intracellular stores such as the endoplasmic reticulum (ER), continuously stimulating calcium influx, which triggers the transcription of differentiation-related genes such as Involucrin and Loricrin^9,10^.

TLR adaptor interacting with SLC15A4 on the lysosome (TASL) localizes with endo-lysosomal TLR7/8/9 and directly binds to solute carrier family 15 member 4 (SLC15A4). SLC15A4, which acts as a proton pump, is involved in lysosomal acidification and is associated with disrupted lysosomal pH regulation depending on TASL expression^11^. TASL contains a conserved pLxIS motif, which mediates the recruitment and activation of the interferon regulatory factor 5 (IRF5) through the activation of endosomal TLRs (TLR7, TLR8, TLR9)^12^. Previous studies have revealed that TASL interacts with SLC15A4, regulating the pH of lysosomes. The pH of lysosomes is known to activate enzymes within them, promoting the autophagy process and facilitating protein degradation, which is essential for cellular function^13^. Specifically, in the process of keratinocyte differentiation, significant structural remodeling occurs, which is required for the intracellular digestion of proteins and organelles. Therefore, lysosome acidification is essential to activate lysosomal enzymes and facilitate autophagy-mediated organelle clearance in keratinocytes^14,15^.

However, there is no research on the role of TASL in keratinocytes. To investigate the function of TASL in keratinocytes, we utilized a TASL knockout (KO) human keratinocyte cell line. TASL KO keratinocytes exhibited inhibited proliferation and abnormal differentiation. Moreover, we detected a significant increase in intracellular calcium levels, which is known to regulate the keratinocyte differentiation process. To the best of our knowledge, this study is the first to investigate the impact of TASL on the differentiation process of keratinocytes.

## Results

### TASL deficiency inhibits the proliferation and migration of HaCaT cells by inducing G1/S arrest

**Figure 1:**
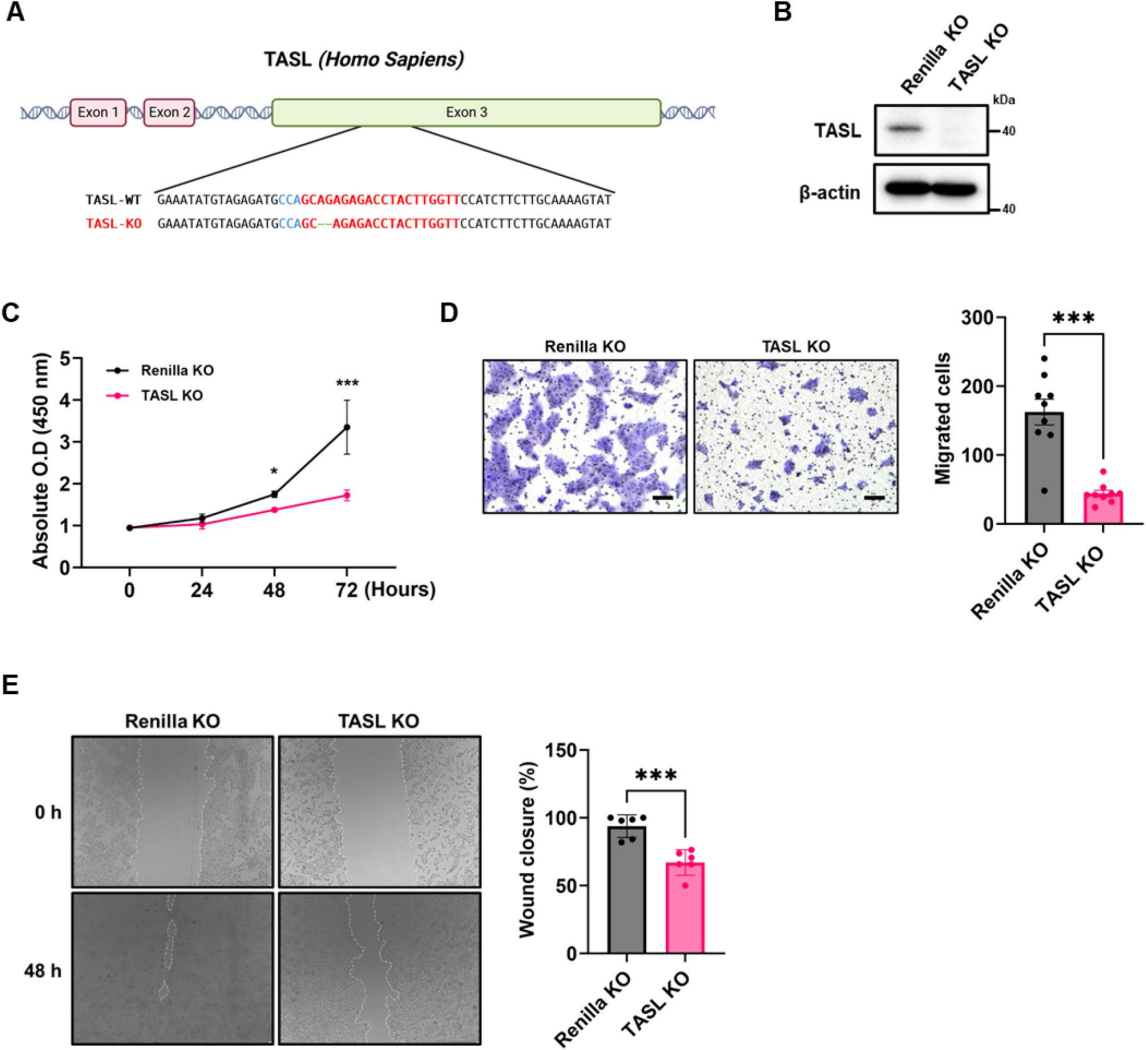
The effect of TASL deletion on the proliferation and migration of HaCaT cells. (**A**) A schematic representation of the TASL gene in TASL KO cells with a deletion of two base pairs in the third exon. This image was created with BioRender.com. (**B**) TASL protein expression levels were confirmed using western blot. (**C**) The proliferation of TASL KO cells was evaluated using a CCK-8 assay at 0, 24, 48, and 72 h. (**D**) The migratory abilities of Renilla KO and TASL KO cells were measured using transwell assay. After adding 10% FBS to the bottom wells, the cells were incubated for 24 h. The migrated cells were stained with 0.1% crystal violet and counted. (**E**) After creating the scratch, the percent of wound closure (%) was measured using ImageJ in Renilla KO and TASL KO cells 48 h later. The wounded area was delineated with a white dashed line. The data are presented as the mean ± SD, and statistical comparisons were performed using two-way ANOVA, student’s t-test. *p < 0.05; ***p < 0.001.

**Figure 2:**
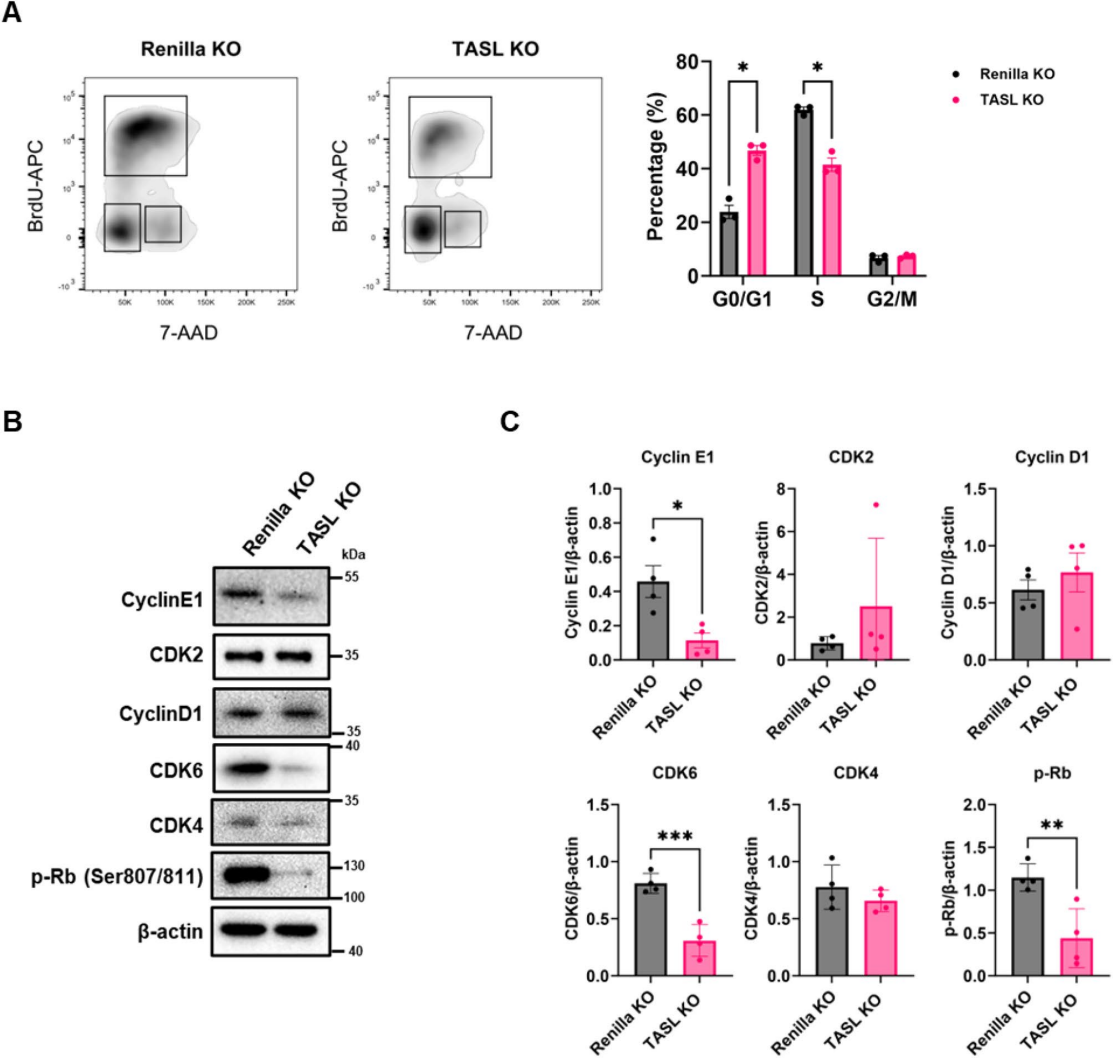
Deletion of TASL induces G1/S cell cycle arrest in HaCaT cells. (**A**) Cell cycle distribution was determined using flow cytometric analysis in Renilla KO and TASL KO cells. The bar graph shows the percentage of Renilla KO and TASL KO cells in G1, S, and G2/M phase. (**B**) The protein expression that induces the transition from the G1 phase to the S phase was confirmed through western blot. (**C**) The expression of cell cycle regulatory proteins was determined by western blot and quantified using ImageJ. The data are presented as the mean ± SD, and statistical comparisons were performed using multiple t-test, and student t-test; *p < 0.05; **p < 0.01; ***p < 0.001.

HaCaT cells are immortalized human keratinocytes widely used for studying epidermal homeostasis. To investigate the effects of TASL deficiency on certain characteristics that influence epidermal homeostasis in keratinocytes, such as proliferation, migration, and differentiation, we established TASL KO HaCaT cells using a lentiCRISPR v2 vector. The target sequences were successfully deleted, resulting in the removal of two base pairs, as confirmed through Sanger sequencing (Fig. 1A). After establishing a stable cell line, the protein expression levels of TASL were assessed using western blot (Fig. 1B). The validation of the custom TASL antibody was confirmed (Supplementary Fig. 1). Proper proliferation and migration are required for the re-epithelialization of skin wounds and the restoration of the epidermal barrier^16^. We initially performed a CCK-8 assay and found that proliferation was significantly decreased in TASL KO cells (Fig. 1C). When using shRNA targeting TASL, we observed a similar trend (Supplementary Fig. 2C). In addition, the number of cells migrating through the transwell membrane in response to FBS was significantly reduced in TASL KO cells, indicating that TASL deficiency results in a decrease in migratory ability (Fig. 1D). To assess the impact of decreased proliferation and migration abilities, a wound healing assay was performed. The wound closure percentage of TASL KO cells was reduced compared to the Renilla KO control (Fig. 1E). Cell cycle deregulation is associated with aberrant cell proliferation^17^. Therefore, we analyzed cell cycle progression to investigate the effect of TASL on keratinocyte proliferation. In keratinocytes, the cell cycle is mainly regulated in the G1 phase. The G1/S transition is regulated by critical cyclins (Cyclin D1 and Cyclin E1) and cyclin-dependent kinases (CDK2, CDK4, and CDK6)^18^. Without any stimulation, the proportion of TASL KO cells in the S phase exhibited a significant reduction (Fig. 2A). To further analyze cell cycle progression, we examined the expression levels of proteins involved in the G1/S transition using western blot. The expression of CDK6 and Cyclin E1 significantly decreased in TASL KO cells. Furthermore, the phosphorylation of Rb by the cyclin-CDK complex, which is responsible for S phase progression, also notably decreased (Fig. 2B,C). These changes indicate that the deletion of TASL inhibits the proliferation and migration of HaCaT cells by inducing G1/S cell cycle arrest.

### TASL deficiency affects the expression of differentiation-related genes in HaCaT cells

**Figure 3:**
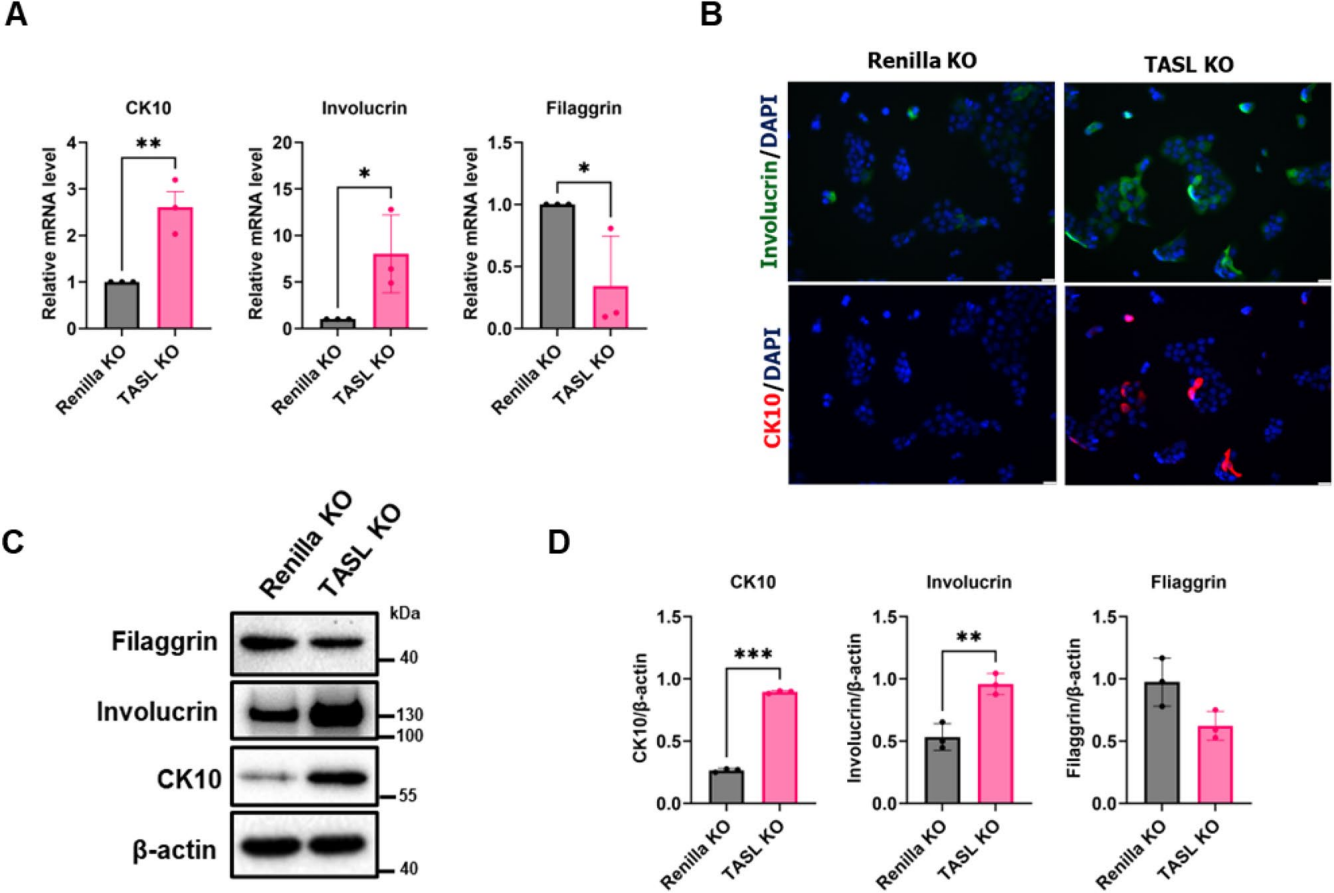
Assessment of the expression of differentiation-related genes in TASL KO HaCaT cells. (**A**) Expression levels of differentiation-related genes, CK10, involucrin, and filaggrin, were measured using qPCR. (**B**) CK10 (red) and involucrin (green) cells were assessed using immunocytochemistry. Scale bar, 50 µm. (**C**) Protein expression levels of CK10, involucrin, and filaggrin were measured using western blot. (**D**) The protein expression of CK10, involucrin, and filaggrin were quantified and normalized with β-actin. The data are presented as the mean ± SD, and statistical comparisons were performed using student’s t-test; *p < 0.05; **p < 0.01; ***p < 0.001.

To investigate the role of TASL in keratinocyte differentiation, we measured the expression levels of differentiation-related genes (CK10, involucrin, and filaggrin) through qPCR. Early differentiation-related genes, CK10 and involucrin, were significantly upregulated, while the late differentiation-related gene filaggrin was downregulated (Fig. 3A)^19,20^. Next, we characterized the expression and localization of CK10 and involucrin using immunofluorescent staining. Involucrin is a soluble protein precursor of the cornified envelope that is synthesized during the early stage of terminal differentiation^20,21^. Involucrin-positive cells were tightly packed and had altered morphology with a wider range and higher expression in TASL KO cells compared to Renilla KO cells. Additionally, TASL KO cells showed a higher number of CK10-positive cells (Fig. 3B). Furthermore, the protein expression levels of CK10 and involucrin significantly increased in TASL KO cells (Fig. 3C,D). The shTASL cell line also exhibited excessive early differentiation compared to the scramble cell (Supplementary Fig. 2D). These data suggest that excessive early differentiation occurs in TASL KO cells, indicating the involvement of TASL in the differentiation process.

### TASL regulates the process of keratinocyte differentiation by maintaining appropriate levels of intracellular calcium

**Figure 4:**
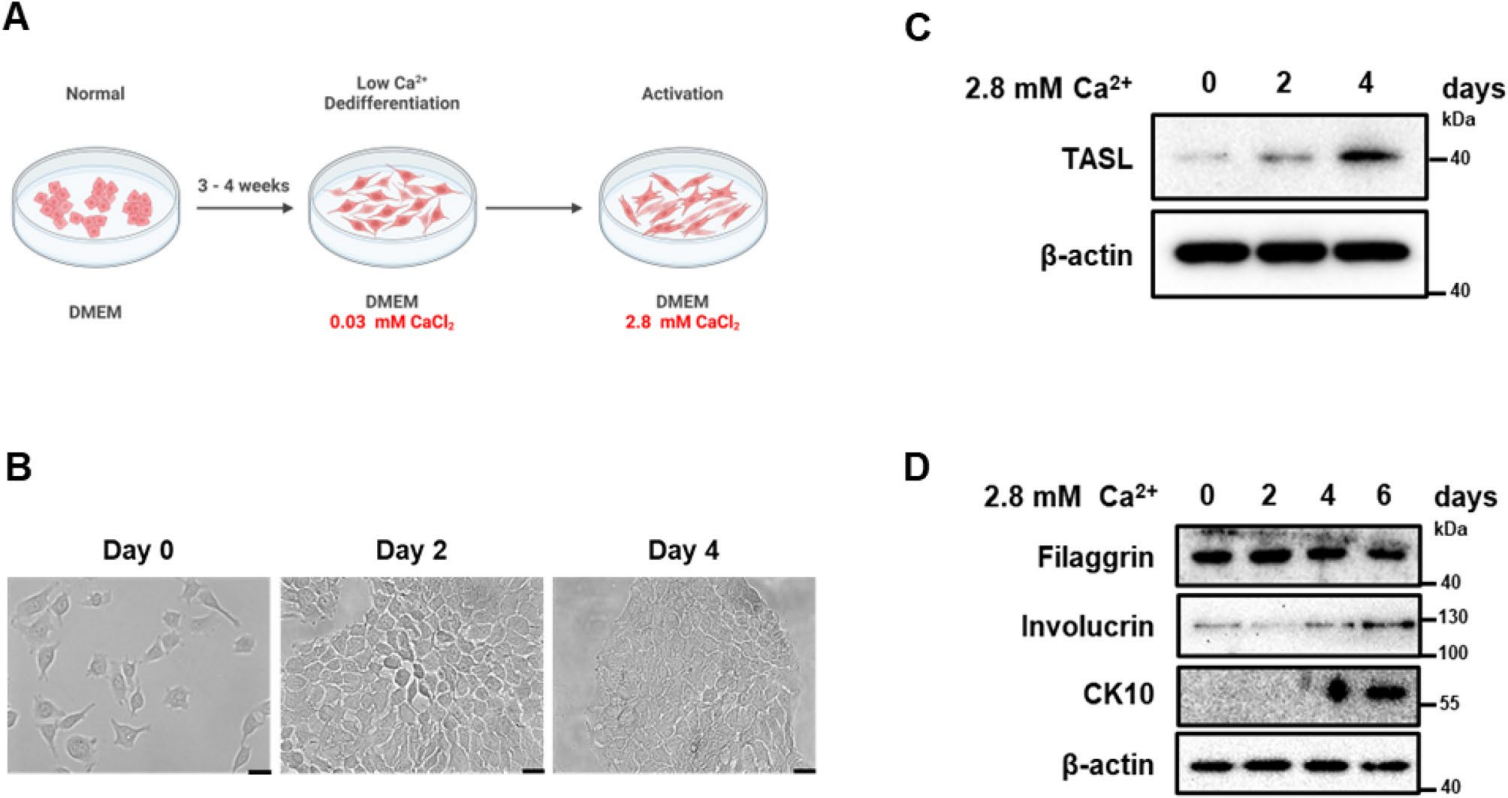
The increase of TASL expression during calcium-induced differentiation. (**A**) Renilla KO and TASL KO HaCaT cells were cultured as illustrated (see the text for details). This image was created with BioRender.com. (**B**) Morphology of HaCaT cells cultured in low calcium conditions for 3–4 weeks. Morphological changes of HaCaT cells induced to differentiation for 2 and 4 days through calcium treatment. Scale bar, 50 µm. (**C**) The expression level of TASL stimulated with calcium (2.8 mM, 0–6 days) was confirmed. (**D**) Calcium-induced differentiation was confirmed through western blot analysis. The protein levels of CK10, involucrin, and filaggrin were measured.

**Figure 5:**
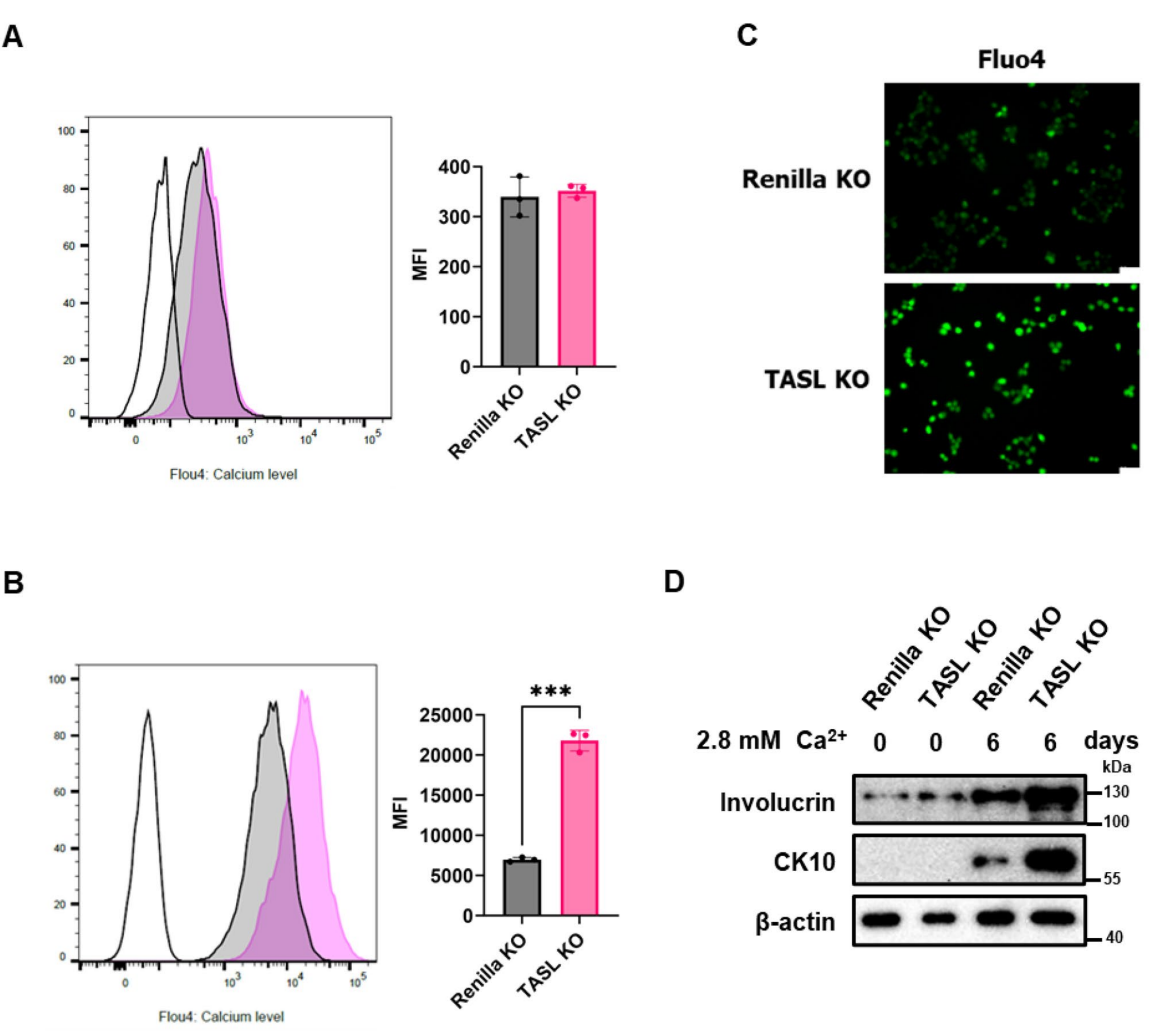
TASL affects intracellular calcium levels in response to an increase in the extracellular calcium in HaCaT cells. (**A**) Intracellular calcium levels were measured in HaCaT cells cultured in low-calcium conditions using FACS. (**B**) Intracellular calcium levels were measured in HaCaT cells cultured in low-calcium conditions using FACS. White line represents the values of the unstained cell, the gray line represents the Renilla KO cell and the pink line represents the TASL KO with fluo4 staining. (**C**) Intracellular calcium concentration was examined using a fluorescence microscope. Scale bar, 50 µm. (**D**) Dedifferentiated Renilla KO and TASL KO cells were induced differentiation with 2.8 mM calcium media for 6 days. The protein expression of CK10 and involucrin were measured using western blot. The data are presented as the mean ± SD, and statistical comparisons were performed using student’s t-test; ***p < 0.001.

Calcium plays a crucial role in regulating the expression of keratinocyte differentiation genes^22^. An increase in extracellular calcium levels leads to a corresponding increase in cytosolic calcium levels and consequently induces differentiation^22,23^. As shown in Fig. 3, an increase in differentiation markers was confirmed, prompting us to investigate whether this was associated with a change in calcium levels. To examine the role of TASL in the regulation of intracellular calcium levels in HaCaT cells, we manipulated the calcium concentration in the medium. Typically, DMEM contains 2.2 mM calcium, and normal HaCaT cells cannot exhibit basal cell properties under this condition. Therefore, we dedifferentiated HaCaT cells into a basal phenotype by culturing the cells in a low-calcium medium containing 0.03 mM calcium, as described in a previous study^24^. The process of dedifferentiation and differentiation through calcium is illustrated in Fig. 4A. To further assess the relationship between TASL and calcium-induced differentiation, we induced differentiation by incubating the cells in media with 2.8 mM calcium for 0, 2, 4, and 6 days. When cultured in low calcium conditions, dedifferentiated HaCaT cells exhibit a spindle-shaped morphology and loosely packed structure. However, induction of differentiation by adding 2.8 mM calcium results in a cuboidal shape and tightly packed structure (Fig. 4B). This indicates that when cultured under low calcium conditions, the cells exhibit a morphology closer to the basal state, suggesting dedifferentiation, while they adopt a differentiated morphology when induced with 2.8 mM calcium. High calcium treatment for inducing differentiation triggered the increase of TASL expression (Fig. 4C). It was confirmed that the expression of differentiation markers, CK10 and involucrin, increased in a time-dependent manner with calcium treatment (Fig. 4D). These findings suggest that TASL is involved in the differentiation process of keratinocytes through calcium. To assess the impact of TASL knockout on intracellular calcium, we stained the cells with fluo4 and measured intracellular calcium levels using flow cytometry and fluorescence imaging. Under low-calcium culture conditions, no changes were observed in intracellular calcium concentration (Fig. 5A). However, under high-calcium culture conditions, TASL KO cells exhibited a significant increase in intracellular calcium levels (Fig. 5B,C). Furthermore, upon induction of differentiation with 2.8 mM calcium, we observed a sharp increase in the expression of early differentiation markers CK10 and involucrin when TASL was deficient (Fig. 5D). This data suggests that TASL may play a role in preventing excessive intracellular calcium accumulation and, in turn, potentially contribute to the differentiation of keratinocytes.

### TASL modulates lysosome biogenesis and lysosomal pH

**Figure 6:**
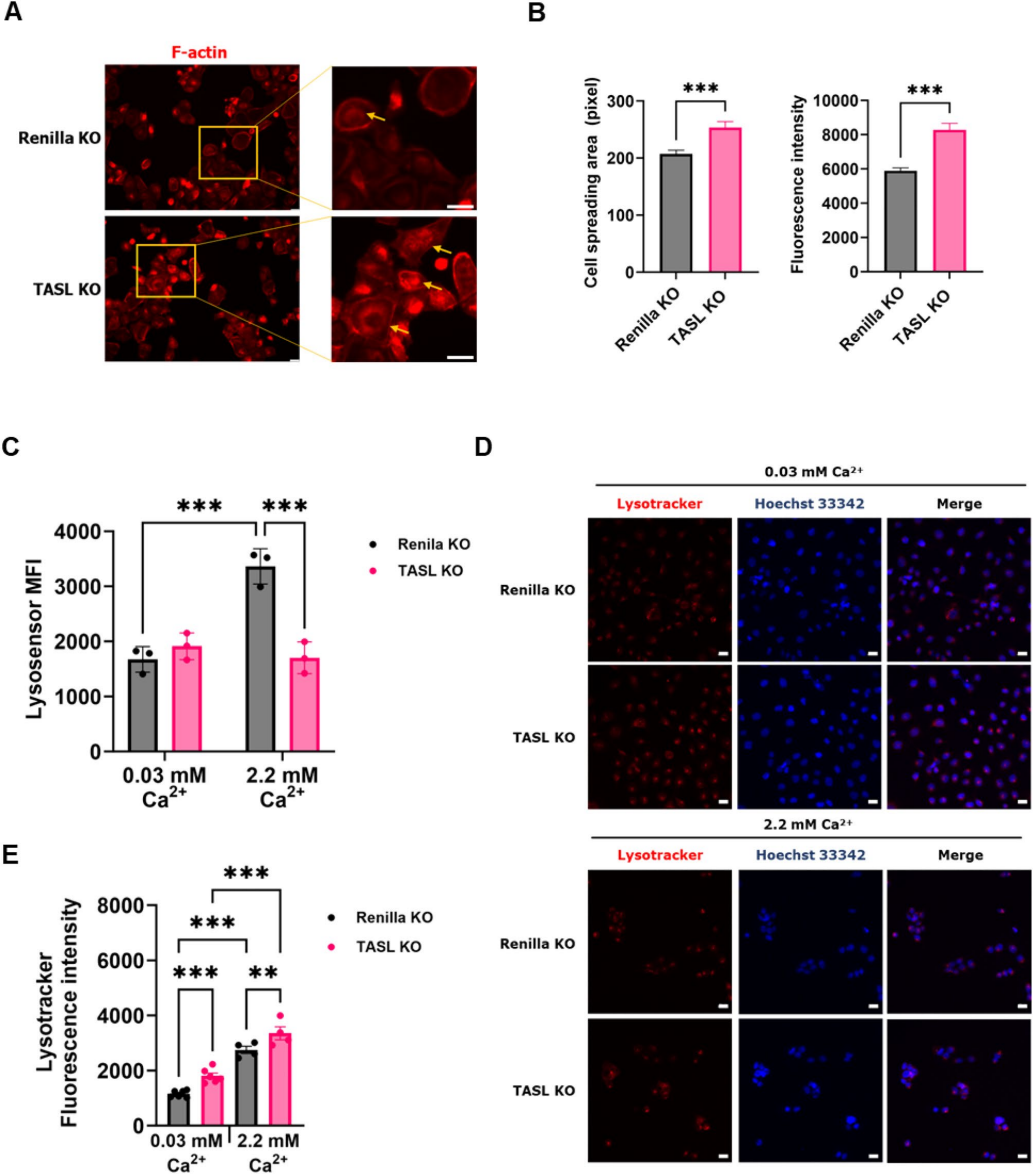
The changes in lysosome biogenesis and lysosome acidification in TASL KO HaCaT cells. (**A**) F-actin (red) was stained in Renilla KO and TASL KO cells grown in low-calcium media, and their morphology and stress fibers were examined. Yellow arrows represent stress fibers. Scale bar, 50 µm. (**B**) Cells exhibiting firmly attached and widely spread-out morphology resembling basal phenotype were counted. Cell size and fluorescence intensity were quantified using ImageJ. (**C**) The cells were treated with LysoSensor (4 µM) for 1 h and analyzed for fluorescence intensity using FACS. (**D**) The cells were treated with LysoTracker Deep Red (75 nM) for 1 h and counterstained with Hoechst 33342 (blue) observed using confocal microscope. Scale bar, 20 µm. The data are presented as the mean ± SD (**B**,**C**) or the mean ± SEM (**E**), and statistical comparisons were performed using student’s t-test and two-way ANOVA; **p < 0.01; ***p < 0.001.

Actin organization occurs during the differentiation of keratinocytes and leads to dynamic changes in cell shape, cell motility, and cell-cell junctions^25,26^. The disassembly and assembly of F-actin for cytoskeleton remodeling not only impact cell shape but also possesses the ability to facilitate the transport of specific molecules crucial for signal transduction^27^. To perform various physiological functions of lysosomes, it is necessary to regulate the movement and positioning of lysosomes through the modulation of the actin cytoskeleton^28^. Thus, we examined whether actin organization was altered and stress fibers were formed by staining filamentous actin (F-actin). In low calcium media, TASL KO cells exhibited a larger and more robust morphology, with a noticeable increase in stress fibers (Fig. 6A,B). To evaluate the changes in lysosome function mediated by TASL during keratinocyte differentiation, we measured lysosomal pH using LysoSensor. Upon calcium-induced differentiation, TASL KO cells showed a significant increase in lysosomal pH compared to the control group (Fig. 6C). Lysosome biogenesis is essential for resolving cellular stress and facilitating cellular adaptation to stimuli^29^. Previous studies have shown that autophagy influx and lysosome biogenesis are upregulated during calcium-induced keratinocyte differentiation^30^. In line with these findings, we observed an upregulation of lysosome biogenesis in TASL KO cells as evidenced by using Lysotracker. As extracellular calcium levels increased, there was a corresponding increase in lysosome biogenesis, which was more pronounced in TASL KO cells (Fig. 6D,E). These results suggest that TASL influences lysosome biogenesis and lysosomal pH during the differentiation process of keratinocytes.

## Discussion

The epidermis, which is the outermost layer of the skin, is composed of keratinocytes^2^. Maintaining the tight balance of keratinocyte proliferation and differentiation is crucial for the homeostasis of the epidermis, directly affecting its structure and function. However, the precise mechanisms by which proliferating cells cease proliferation, migrate, and undergo differentiation are not completely understood.

As keratinocytes differentiate, they undergo an increase in cell size and an augmentation in the number of cell organelles^31^. Recent findings have shown that lysosomes play a crucial role in normal epidermal differentiation by regulating differentiation-related signaling pathways and degrading increased cellular organelles within keratinocytes during the differentiation process^13^. TASL is a specific interaction partner of SLC15A4, which modulates lysosomal pH and lysosome biogenesis^11,32^. Lysosomes are involved in protein degradation and signaling coordination^13^. Lysosomal acidification tightly regulates lysosomal function by activating enzyme activity, promoting autophagosome formation, and facilitating protein degradation^33^. Lysosome biogenesis is essential for resolving cellular stress and supporting cellular adaptation to stimuli^29^. Recent findings suggest that lysosomes regulate signaling and degradation necessary for normal epidermal differentiation. However, the role of TASL in keratinocytes is poorly understood.

To investigate the role of TASL in the formation of the epidermal barrier by keratinocytes, we established a TASL KO HaCaT cell line. Interestingly, TASL KO cells showed reduced proliferation by inducing G1/S cell cycle arrest (Figs. 1C, 2) and highly increased expression of differentiation-related proteins (Fig. 3C,D). The regulation of the cell cycle progression is a prominent mechanism governing cellular growth. In keratinocytes, multiple factors influence the progression of the cell cycle, including the level of differentiation, the degree of matrix adhesion, and the effects of growth factors^34^.

The proliferation and differentiation of keratinocytes are closely related and regulated by various factors. Among these factors, calcium is extensively researched and known to be a crucial most crucial inducer of differentiation in keratinocytes^22^. An increase in external calcium levels activates the release of calcium from internal cell organelles such as the ER, lysosomes, and Golgi apparatus, leading to differentiation^8,22^. As the concentration of calcium increases, it initiates terminal differentiation, thereby inhibiting proliferation.

Interestingly, TASL expression showed a gradual increase over time as keratinocyte differentiation was induced with calcium (Fig. 4B,D). Alongside this TASL increment, there was a rapid increase in Involucrin and CK10 expression but minimal change in processed filaggrin expression (Fig. 4D). While there have been debates about the role of filaggrin in the differentiation of HaCaT cells, its expression was hardly altered during calcium-induced differentiation^35^. These observations imply that TASL might be involved in keratinocyte differentiation through calcium signaling. Also, it is worth considering that TASL could influence proliferation as well, by inducing differentiation via calcium while concurrently inhibiting proliferation.

Moreover, it was possible to confirm the association of TASL with calcium regulation and changes. When external calcium levels were increased, a significant elevation in intracellular calcium concentration was observed in correlation with TASL deficiency. When the expression of the differentiation markers increased (Fig. 3), concurrent with the elevated external calcium, TASL KO cells were observed to have a higher intracellular calcium content (Fig. 5). The factors regulating intracellular calcium levels have complex mechanism involving ion channels and pumps on the cell membrane, calcium recognition via ligand-receptor interactions, and release from intracellular stores such as the lysosomes, ER, and Golgi apparatus, as well as intracellular signaling pathways^8,10^. Although the mechanism underlying the regulation of calcium levels by TASL is unclear, these findings provide evidence that TASL not only regulates differentiation but also participates in modulating calcium levels.

Calcium has long been recognized as the primary factor influencing keratinocyte differentiation^22^. However, recent studies have shown that impaired lysosomal function prevents proper differentiation when extracellular calcium is increased, underscoring the significance of lysosomal function in this process^13^. Lysosomes are crucial cellular organelles responsible for storing intracellular calcium^36,37^. An increase in calcium ion levels prompts the release of calcium from lysosomes, subsequently triggering lysosome biogenesis and activating autophagy-related genes^38^. The precise mechanisms governing the regulation of calcium levels and pH within lysosomes have been challenging to confirm^39^. Nevertheless, when TASL was knocked out, proper acidification within lysosomes did not occur despite the increases in extracellular calcium levels. However, an increase in lysosome biogenesis was observed in TASL KO cells (Fig. 6D,E). Therefore, the deficiency of TASL, resulting in lysosomal dysfunction, is associated with the inability to trigger proper differentiation despite increased calcium levels. Additionally, the disruption of lysosomal function may compromise calcium homeostasis, potentially leading to detrimental calcium release.

In summary, this study examined the association between TASL and keratinocyte function *in vitro*. Our data confirmed that a deficiency of TASL impairs lysosomal function and triggers the dysregulation of calcium, leading to the inhibition of proliferation, migration, and early differentiation in keratinocytes. The inhibition of proliferation and migration prevents proper wound healing after injury. Moreover, excessive early differentiation induces abnormal differentiation, disrupting epidermal homeostasis. Future studies are needed to determine the precise mechanisms that regulate these molecular processes. Still, this study provides a foundation for understanding the multifaceted involvement of TASL in keratinocyte function and suggests its broader implications in therapeutic interventions beyond skin disorders.

## Materials and methods

### Cell culture and transfection

The HaCaT cells, a human epidermal keratinocyte cell line, were purchased from AddexBio Technologies (San Diego, CA, USA) and cultured in Dulbecco’s Modified Eagle’s Media (DMEM, Gibco) supplemented with 10% Fetal Bovine Serum (FBS, Gibco) and 1% Penicillin-Streptomycin (P/S, Gibco) at 37 °C with 5% CO_2_. To establish stable TASL knockout (KO) cells, HEK293T cells were cotransfected with lentiCRISPR v2 with sgRNA targeting the third exon of human TASL or Renilla lucifease and pMDLg/pRRE, pMD2.G, and pRSV-Rev using FuGENE HD transfection reagent (Promega, USA). The virus was harvested 48 h after transfection and filtered through a 0.45 μm filter. HaCaT cells were tranduced with lentiviruses encoding sgRNA targeting human TASL or Renilla luciferase. The control sgRNA targeting Renillla luciferase (sgRenilla): forward, 5′-CACCGGTATAATACACCGCGCTAC-3′ reverse, 5′-AAACGTAGCGCGGTGTATTATACC-3′. The human TASL sgRNA sequence: forward, 5′-CACCGAACCAAGTAGGTCTCTCTGC-3′; reverse, 5′-AAACGCAGAGAGACCTACTTGGTTC-3′. After transduction, single-cell colonies were established in 96-well plates. The protein expression levels of TASL were examined in the expanded clones. To assess the mutations and deletions of TASL in these clones, the target sequence was analyzed using Sanger Sequencing.

### Cell counting Kit-8 assay

To examine the proliferation of the HaCaT cell line, the CCK-8 assay (Dojindo, MD, USA) was used. A total of 1 × 10^3^ HaCaT cells were seeded into 96-well plates and incubated for 0, 24, 48, and 72 h at 37 °C with 5% CO_2_. Then, 10 µL of CCK-8 solution was added to each well, and the cells were incubated for 2 h. The absorbance was measured at 450 nm using a SPECTROstar Nano device (BMG LABTECH, Germany).

### Wound healing assay

Renilla KO and TASL KO HaCaT cells were plated at a density of 5 × 10^5^ in a 12-well plate. To evaluate cell migration, the culture medium was removed, and the cell layer was scratched to create a wound using a 200-µL pipet tip. To remove the scratched cells, the cells were washed three times using DPBS. Subsequently, DMEM supplemented with 5% FBS and 1% P/S was added, and the cells were incubated for 48 h. The area of the scratch was measured using the MRI Wound Healing Tool available in the ImageJ software (https://github.com/MontpellierRessourcesImagerie/imagej_macros_and_scripts/wiki/Wound-Healing-Tool).

### Transwell Migration assay

Renilla KO and TASL KO HaCaT cells were seeded at a density of 3 × 10^4^ in starved medium onto the upper well of transwell with polycarbonate membrane and 8-μm pores (CLS3422, Corning, USA). After 24 hours, 10% FBS containing DMEM was added to the lower well of the transwell and cultured for another 24 hours. Cells that had migrated through the membrane to the lower chamber were fixed in methanol, stained with 0.1% crystal violet, and washed with PBS. The remaining cells in the upper chamber were gently removed using a cotton swab. The cells that migrated to the underside of the membrane were captured using the microscope and counted using ImageJ.

### BrdU cell cycle analysis

Renilla KO and TASL KO HaCaT cells were plated at a density of 5 × 10^5^ in a 6-well plate. For the cell cycle analysis, BrdU flow kits (552598, BD Bioscience, USA) were used. Renilla KO and TASL KO HaCaT cells were incubated in 10 µM BrdU in growth medium for 20 min. After incubation, cells were harvested and fixed in 1x BD Cytofix/Cytoperm Buffer. The fixed cells were washed in 1x BD Perm/Wash buffer, resuspended in BD Cytoperm Permeabilization Buffer Plus, and incubated for 10 min on ice. Subsequently, the cells were washed with 1xBD Perm/Wash buffer, resuspended in 100 µL Cytofix/Cytoperm Buffer for 5 min, washed once again with 1xBD Perm/Wash buffer, and treated with diluted DNase for 30 min. The cells were washed and incubated with anti-BrdU-FITC antibody (20 min, room temperature). Following the incubation, the cells were washed with 1 mL of 1x BD Perm/Wash buffer and resuspended in 20 µL of the 7-aminoactinomycin (7-AAD) solution. The DNA content was analyzed using a BD FACSVerse Flow Cytometer and FlowJo Software (BD Biosciences, San Jose, CA).

### LysoSensor pH and lysosome imaging

Renilla KO and TASL KO cells were treated with 4 µM LysoSensor Green DND-189 (Invitrogen, USA) at 37 °C for 1 h in a growth medium and washed with DPBS. The cells were harvested, and the fluorescence intensity was analyzed using a BD FACSVerse Flow Cytometer and FlowJo Software (BD Biosciences, San Jose, CA). To confirm lysosome identity, the cells were incubated with 75 nM LysoTracker Deep Red, Carl Zeiss, Germany). Images were captured immediately using a LSM 800 with Airy Scan (Carl zeiss, Germany).

### Keratinocyte differentiation

For keratinocyte differentiation, HaCaT cells were seeded into 12-well plates and grown in DMEM with 0.03 mM CaCl_2_ for 3–4 weeks to achieve an undifferentiated cell condition. The HaCaT cells were cultured in calcium-free DMEM (2068028, Gibco, USA) supplemented with 10% chelated FBS. FBS was calcium-depleted with Chelex 100 (C7901, Sigma-Aldrich, Germany). The cells were subcultured every 3–4 weeks in low-calcium media to maintain their characteristics. To initiate the differentiation process, 2.8 mM calcium was added to DMEM supplemented with 0.03 mM CaCl_2_ for 0, 2, 4, and 6 days.

### Measurement of intracellular calcium levels

HaCaT cells were seeded in 12-well plates and incubated at 37 °C with 5% CO_2_. To measure the levels of intracellular free calcium, the cells were treated with 2 µM Fluo-4 AM (Cayman, USA) in DPBS. The plates were incubated in the dark for 30 min. After the incubation period, the cells were washed three times with DPBS and collected using 0.25% trypsin EDTA (Gibco, USA). The total fluorescence was observed using a fluorescence microscope, and the fluorescence intensity was analyzed using a BD FACSVerse Flow Cytometer and FlowJo Software (BD Biosciences, San Jose, CA).

### Immunocytochemistry (ICC)

Renilla KO and TASL KO cells were seeded into 12-well plates. ICC was performed using antibodies against CK10 (Abcam, ab76318) and Involucrin (Santa Cruz, sc-21748). The cells were then incubated with anti-rabbit IgG conjugated with Alexa Fluor 555 (Thermo Scientific, A-21428) and anti-mouse IgG Alexa Fluor 488 (Thermo Scientific, A28175). Alexa Fluor 568 Phalloidin (Thermo Scientific, A12380) was used to visualize actin polymerization in control and TASL KO cells grown in low-calcium conditions. The cells were observed using a fluorescence microscope (Leica DMI3000B, Leica, Germany).

### Reverse transcription followed by quantitative PCR

Total RNA was isolated from the Renilla KO and TASL KO HaCaT cells using TRIzol (Invitrogen) according to the manufacturer’s instructions. The cDNA was synthesized using a PrimeScript 1st strand cDNA Synthesis Kit (TAKARA), and the mRNA expression levels were measured using the TB Green Advantage qPCR Premix (TAKARA) on a Light Cycler 96 System. The mRNA expression levels of keratin 10 (CK10), involucrin, filaggrin, and β-actin were measured. The β-actin gene was used as an internal control for quantification. The primer sequences used are included in Supplementary Data 1.

### Immunoblotting analysis

Total protein was extracted from Renilla KO and TASL KO HaCaT cells using PRO-PREP lysis buffer (iNtRON Biotechnology) containing Xpert phosphatase inhibitor cocktail (Genedepot, USA), following the manufacturer’s instructions. Protein concentration was measured using a Nanodrop 2000 spectrophotometer (Thermo Scientific). An equal amount of protein obtained from each sample was subjected to electrophoresis using 10% sodium dodecyl sulfate-polyacrylamide gels. After electrophoresis, the proteins were transferred onto 0.45 µm PVDF membranes (Cytiva, USA), which were then blocked in 5% nonfat milk (MB Cell, Los Angeles, CA, USA) or 5% BSA (GenDEPOT, USA) for 1 h. The blots were cut prior to hybridization with antibodies during the blotting. The membranes were incubated with primary antibodies at 4 °C overnight. After washing, the membranes were incubated with secondary antibodies for 2 h at room temperature.

The following primary antibodies were used in this study: custom made TASL (1:1000, Thermo Scientific, information described in supplementary Data 2), CK10 (1:50000, Abcam, ab76318), Filaggrin (1:1000, Santa Cruz Biotechnology, sc-66192), Involucrin (1:1000, Santa Cruz Biotechnology, sc-21948), Cyclin E1 (1:1000, Cell Signaling Technology, 20808s), CDK2 (1:1000, Cell Signaling Technology, 2546S), CDK4 (1:1000, Santa Cruz Biotechnology, sc-70831), CDK6 (1:1000, Cell Signaling Technology, 3136p), Cyclin D1 (1:1000, Cell Signaling Technology, 2922), p-Rb (1:1000, Cell Signaling Technology, 9308), and β-actin (1:10000, Santa Cruz Biotechnology, sc-47778). The following secondary antibodies were used: anti-mouse IgG (1:10000, Thermo Scientific, 62-6520) and anti-rabbit IgG (1:10000, Cell Signaling Technology, 7074). The protein bands were visualized using West-Q Pico ECL solution (GenDEPOT). The band intensities were normalized to those of β-actin and analyzed using ImageJ.

### Statistical analysis

The data are presented as the mean ± standard deviation (SD) and ± standard error of the mean (SEM). All experiments were performed at least three times. Statistical significance was determined through a two-tailed Student t-test, multiple t-test, and two-way ANOVA using GraphPad Prism version 10.1.0 (GraphPad Software).

### Supplementary Information

Dataset S1.

Dataset S2.

Supplementary Figures.

## Data Availability

The datasets used and/or analyzed during the current study available from the corresponding author on reasonable request.
