# Supplementary material for: TASL mediates keratinocyte differentiation by regulating intracellular calcium levels and lysosomal function
Source: Sci Rep. 2024 May 14;14:10978. doi: 10.1038/s41598-024-61674-3 (PMC11094165; doi:10.1038/s41598-024-61674-3)
Supplement: Supplementary file 2 — Dataset S2. [file 41598_2024_61674_MOESM2_ESM.pdf]

TASL Custom antibody

Host : Rabbit

Clonality : Polyclonal antibody

Affinity Purification : Yes

Antigen : Tasl 243:260

Peptide sequence: EFIMTNVDQISLQVSKEK (18aa)

Concentration : 0.42mg/ml
