# Supplementary material for: TASL mediates keratinocyte differentiation by regulating intracellular calcium levels and lysosomal function
Source: Sci Rep. 2024 May 14;14:10978. doi: 10.1038/s41598-024-61674-3 (PMC11094165; doi:10.1038/s41598-024-61674-3)
Supplement: Supplementary file 3 — Supplementary Figures. [file 41598_2024_61674_MOESM3_ESM.pdf]

TASL mediates keratinocyte differentiation by regulating intracellular calcium levels and lysosomal function

Ji Yeong Park[1,+], Hyeng-Soo Kim[2,+,*], Hyejin Hyung[1,+], Soyeon Jang[1,+], Jiwon Ko[1], Jin Hong Lee[1], Si-Yong Kim[1], Song Park[3], Junkoo Yi[4], Sijun Park[2], Su-Geun Lim[2], Seonggon Kim[5], Sanggyu Lee[1], Myoung Ok Kim[6], Soyoung Jang[1,*], and Zae Young Ryoo[1,*]

[1]School of Life Science, BK21 FOUR KNU Creative BioResearch Group, Kyungpook National University, Daegu, 41566, Republic of Korea

[2]Institute of Life Science and Biotechnology, Kyungpook National University, Daegu 41566, Republic of Korea

[3]Department of Animal Science, Gyeongsang National University, Jinju 52828, Republic of Korea

[4]School of Animal Life Convergence Science, Hankyong National University, Anseong 17579, Korea

[5]Preclinical Research Center, Daegu-Gyeongbuk Medical Innovation Foundation, Daegu, Korea

[6]Department of Animal Science and Biotechnology, Research Institute for Innovative Animal Science, Kyungpook National University, Sangju-si, Gyeongsangbuk-do 37224, Republic of Korea

* wkdthdud21@naver.com

* grayciel@knu.ac.kr

* jaewoong64@knu.ac.kr

[+]these authors contributed equally to this work

Supplementary Figure 1

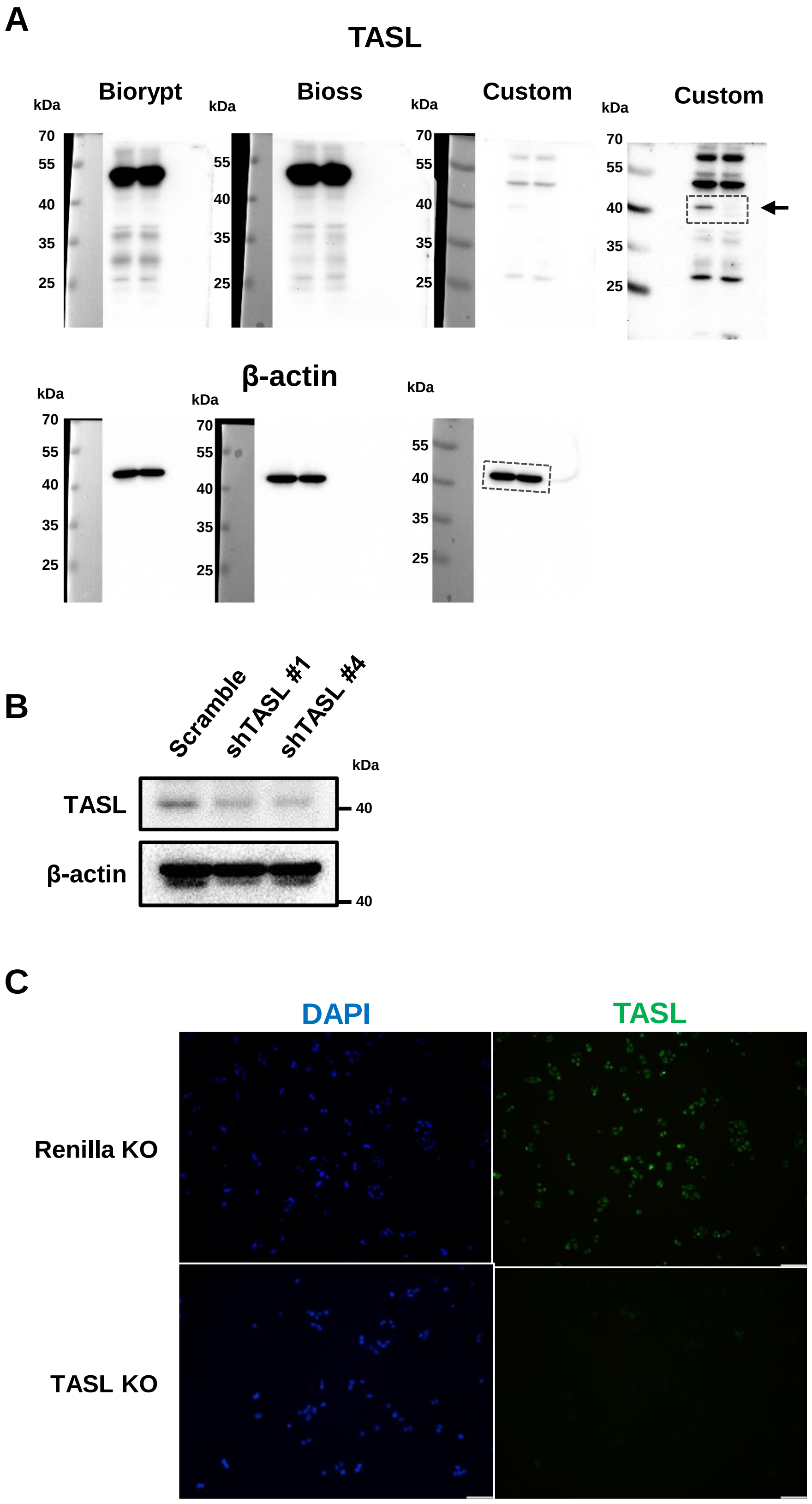

Supplementary figure 2. Confirmation of TASL expression levels using various antibodies.

(A) Immunoblots of Renilla KO and TASL KO HaCaT cells using commercial TASL antibodies (Biorypt, Bioss) and custom-made antibody. (B) Immunoblots of scramble and shTASL transduced HaCaT cells using custom-made antibody. (C) Renilla KO and TASL KO cells stained with custom-made TASL antibody. DAPI (Blue) and TASL (Green). Scale bar, 50 μm.

Supplementary Figure 2

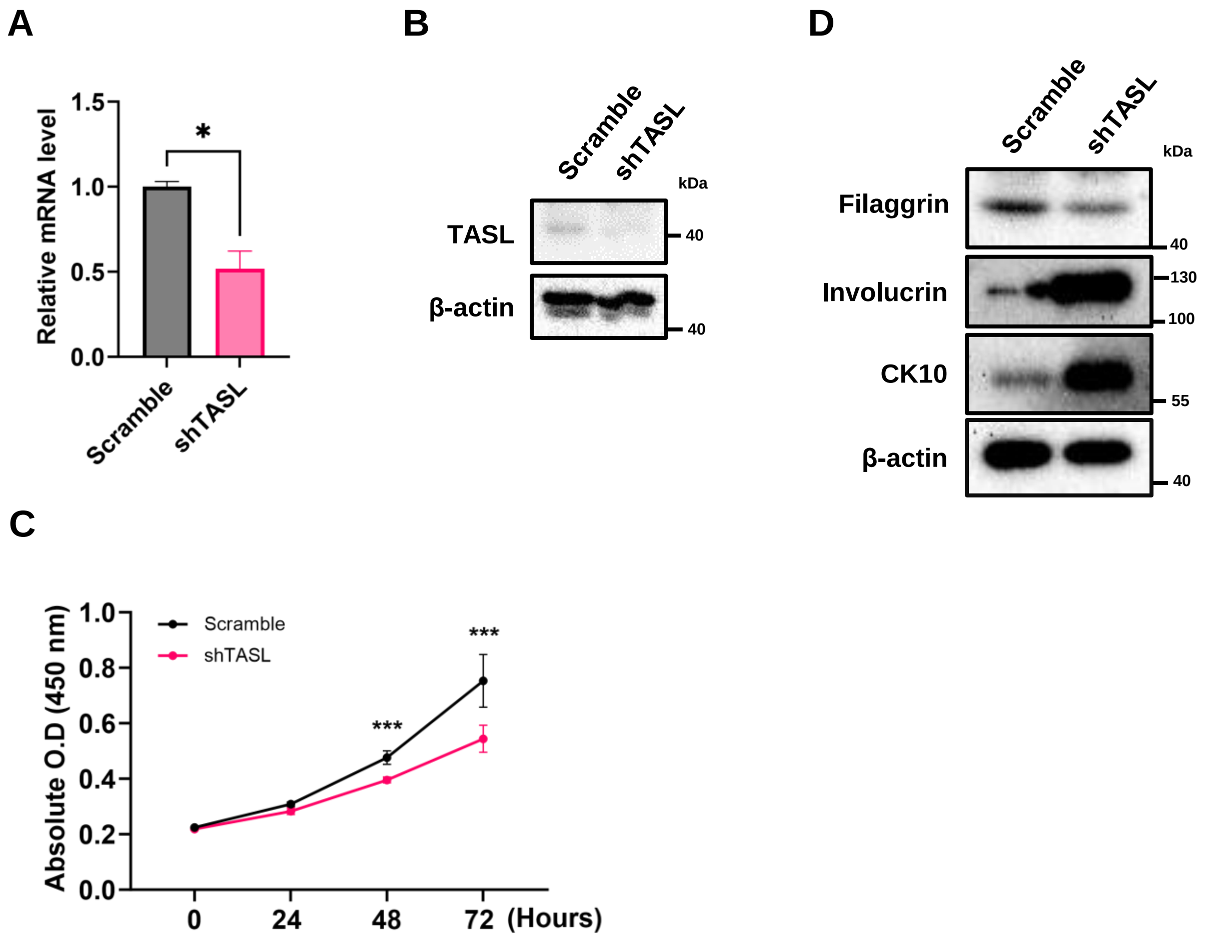

Supplementary figure 2. Characteristics of shTASL HaCaT cells. (A, B) The TASL mRNA and protein expression level in scramble and shTASL transduced HaCaT cells. (C) The proliferation ability of scramble and shTASL HaCaT cells was measured by CCK-8 assay. (D) The protein level of CK10 and Involucrin were increased in TASL knockdown HaCaT cell. The data are presented as the mean ± SD, and statistical comparisons were performed using two-way ANOVA; *, $p < 0.05$; ***, $p < 0.001$.
